# Immune Modulation to Enhance Bone Healing—A New Concept to Induce Bone Using Prostacyclin to Locally Modulate Immunity

**DOI:** 10.3389/fimmu.2019.00713

**Published:** 2019-04-05

**Authors:** Sebastian Wendler, Claudia Schlundt, Christian H. Bucher, Jan Birkigt, Christian J. Schipp, Hans-Dieter Volk, Georg N. Duda, Katharina Schmidt-Bleek

**Affiliations:** ^1^Julius Wolff Institute and Center for Musculoskeletal Surgery, Charité–Universitätsmedizin Berlin, Berlin, Germany; ^2^Berlin-Brandenburg Center for Regenerative Therapies, Charité–Universitätsmedizin Berlin, Berlin, Germany; ^3^Department of Isotope Biogeochemistry, Helmholtz Centre for Environmental Research–UFZ, Leipzig, Germany; ^4^Institute of Medical Immunology, Charité–Universitätsmedizin Berlin, Berlin, Germany; ^5^Berlin Institute of Health Center for Regenerative Therapies, Berlin, Germany

**Keywords:** bone healing, immune modulation, prostacyclin analog, T cell, macrophage, immune cell, Iloprost

## Abstract

Within an aging population, fracture incidences will rise and with the augmented risks of impaired healing the overall risk of delayed bone regeneration will substantially increase in elderly patients. Thus, new strategies to rescue fracture healing in the elderly are highly warranted. Modulating the initial inflammatory phase toward a reduced pro-inflammation launches new treatment options for delayed or impaired healing specifically in the elderly. Here, we evaluated the capacity of the prostacyclin analog Iloprost to modulate the inflammatory phase toward a pro-regenerative milieu using *in vitro* as well as *in vivo* model systems. *In vitro*, Iloprost administration led to a downregulation of potential unfavorable CD8+ cytotoxic T cells as well as their pro-inflammatory cytokine secretion profile. Furthermore, Iloprost increased the mineralization capacity of osteogenic induced mesenchymal stromal cells through both direct as well as indirect cues. In an *in vivo* approach, Iloprost, embedded in a biphasic fibrin scaffold, decreased the pro-inflammatory and simultaneously enhanced the anti-inflammatory phase thereby improving bone healing outcome. Overall, our presented data confirms a possible strategy to modulate the early inflammatory phase in aged individuals toward a physiological healing by a downregulation of an excessive pro-inflammation that otherwise would impair healing. Further confirmation in phase I/II trials, however, is needed to validate the concept in a broader clinical evaluation.

## Introduction

Bone is one of the few tissues in the human body capable of regenerative, scar-free healing. Thus, a bony injury can result in complete restoration of form and function, a restitutio ad integrum. However, the complex bone healing process consisting of sequential, partly overlapping phases is prone to failure (1–4). Even in today's medical routine 5–10% of fracture patients suffer from delayed healing or a resulting non-union (5–7). Therefore, impaired bone repair after injury is still a clinically relevant problem, which will even further increase in the overall aging population (8). Thus, a better and deeper understanding of the underlying biological mechanisms under unimpaired healing conditions is necessary for the development of novel therapeutic treatment strategies to improve unsuccessful bone regeneration.

The tight interaction of the immune system and bone healing has been recognized in the emerging research field of osteoimmunology. Especially the early phase of healing, the inflammatory phase seems to be a promising target for immuno-modulatory approaches to enhance bone healing (9, 10). The pro-inflammatory reaction following injury (11) is an essential trigger or initiator of the healing process. However, a pronounced or prolonged pro-inflammatory reaction (due to a lack or damped anti-inflammatory phase) will negatively impact the healing process (12–14). Recently, specific subsets of the immune system have been shown to negatively influence bone formation: Effector and effector memory CD8+ T cells are producers of TNFα (tumor necrosis factor alpha) and IFNγ (interferon gamma), highly pro-inflammatory cytokines which have been found to deter osteogenic differentiation (15). Therefore, downregulation of the negative influence of immune cell subsets could potentially enhance bone healing. Anti-inflammatory cytokines such as interleukin (IL) 4/IL-13 further the M2/Th2 response, thus promoting the immune response triggered by tissue injury under a regulatory phenotype rather than a M1/Th1 pro-inflammatory phenotype. A proof of concept study showed that applying IL-4/IL-13 during the initial bone healing phase could indeed enhance bone formation (16). However, a distinct initiation of an anti-inflammation has not been evaluated so far.

Within this study, a prostacyclin (PGI_2_) analog was tested as a possible immune modulatory drug to enhance bone formation. PGI_2_ is a small molecule derived from arachidonic acid by cyclooxygenase-2 (Cox-2) and prostacyclin synthase. Endogenous PGI_2_ is already well-known playing an important role in cardiovascular diseases due its vasodilatory function (17, 18). In recent years, a potential role of PGI_2_ as immune modulatory agent was detected by promoting an anti-inflammatory and immunosuppressive effect (19, 20). In particular, the PGI_2_ receptor (IP) is present on platelets, medullary thymocytes, neutrophils, dendritic cells, eosinophils, T regulatory cells, and activated T cells (21, 22). Thus, PGI_2_ has an impact on both, cells of the innate, and of the adaptive immunity. Due to the strong interconnectivity of the immune system and the skeletal system during bone regeneration, PGI2 could represent a potential and promising agent to further bone fracture healing. In the context of bone injuries, the PGI2 analog Iloprost was already successfully used to treat bone marrow edema and avascular necrosis (23, 24). However, the effect of PGI_2_ on the process of bone formation/regeneration was not analyzed by any study so far. In the here presented study, we investigated the immune modulatory effect of PGI_2_ in the context of bone regeneration. Since the half-life of endogenous PGI_2_ is very short, the PGI_2_ analog Iloprost was used. Iloprost is approved as treatment for pulmonary arterial hypertension (25) and peripheral arterial occlusive disease (26), respectively. Within the here presented study, we focused on the immune suppressive capacities of Iloprost, especially on CD8+ T cells and macrophages. Immune modulatory properties were confirmed and the postulated positive osteogenic effect was verified *in vitro*. In a final proof of concept *in vivo* trial, the positive impact of an application of Iloprost during the early bone healing phase was demonstrated in a mouse osteotomy model.

## Materials and Methods

### Animal Model

Female C57BL/6N (Charles River Laboratories, Wilmington, MA, USA) were used for the analysis of the bone healing capacity *in vivo*. All mice were purchased at an age of 8 weeks and mice were housed in small groups in our animal facility. Animals were kept for at least 4 weeks in the non-SPF area of the animal facility (area in the animal facility without filtered air supply for the cages and without additional barrier) to allow a higher environmental pathogen exposure to challenge and to moderately activate the adaptive immune system of the animals. All mice experiments were carried out with the ethical permission according to the principles and policies established by the Animal Welfare Act, the National Institutes of Health Guide for Care and Use of Laboratory Animals, and the National Animal Welfare Guidelines. All animal experiments were approved by the local legal representative animal rights protection authorities (Landesamt for Gesundheit und Soziales Berlin: G0008/12; T0119/14; T0249/11). All results are reported according the ARRIVE guidelines.

### Sample Harvesting for the *in vitro* Analysis With Immune Cells

For the immunomodulatory analysis *in vitro*, femora, and humeri were harvested from C57BL/6 N mice. To isolate the bone marrow, the epiphyses were cut off from the bones and the bone marrow was flashed out into RPMI 1640 media (Biochrom, Berlin, Germany). The bone marrow was pushed through a 40 μm cell strainer to get a single cell suspension. Residing erythrocytes were lysed for 4 min at room temperature (RT) in ACK lysing buffer (Gibco Life Technologies GmbH, Darmstadt, Germany). After centrifugation, cells were resuspended in 10 ml RPMI 1640 media and counted.

### Isolation of CD8+ T Cells

CD8+ T cells were isolated from bones harvested from C57BL/6N mice. The isolation was performed via the *CD8 S-pluriBeads anti-ms* kit (pluriSelect Life Sciences, Leipzig, Germany). The isolation was carried out following the manufacturer's instructions. Briefly, complete bone marrow cells were resuspended in a 1:2 mixture of the isolation and wash buffer and 40 μl S-pluriBeads were added per 1 x 10^6^ target cells and the mixture was incubated for 30 min at RT while continuous slowly shaking (horizontal roller mixer). Cell mixture was washed trough a S-pluriStrainer and target cells remained on the S-pluriStrainer. To detach the CD8+ T cells from the S-pluriBeads, detachment activation buffer D was added to the cells. Detached isolated cells were collected in a new tube, washed, and counted.

The purity of the isolated CD8+ T cells was confirmed by flow cytometry. The following antibodies were used: Life/Dead, α-CD3 PerCP, α-CD4 AF700, and α-CD8 eF450. The incubation with the antibodies was done on ice for 20 min. After the staining, cells were washed, fixed, and analyzed with a flow cytometer LSR II (Becton Dickinson Bioscience, Heidelberg, Germany).

### Study Design for the *in vitro* Analysis of the Osteogenic and Osteoimmunological Effect of Iloprost

**Figure 1:**
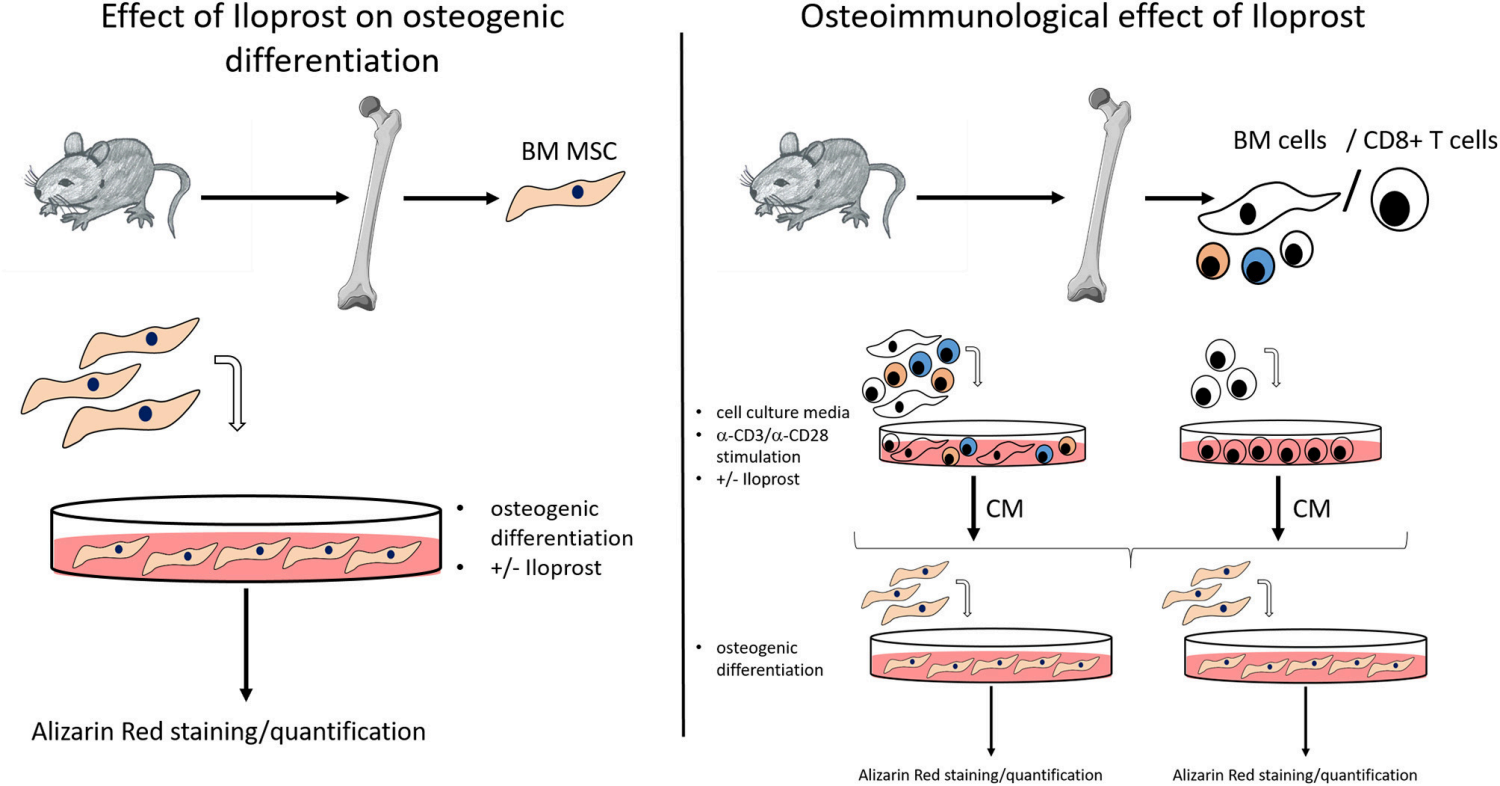
Methodological scheme for the *in vitro* analysis of the osteogenic effect and osteoimmunological effect of Iloprost. BM MSC, bone marrow mesenchymal stromal cells; BM cells, bone marrow cells; CM, conditioned media.

The objective of this study was to investigate the potential of the prostacyclin analoque Iloprost to improve bone healing. For this analysis, the osteogenic and osteoimmunological effect of Iloprost was first evaluated *in vitro*. Subsequently, Iloprost was inserted into a fibrin clot in order to confirm the pro-osteogenic potential of Iloprost in an *in vivo* proof of concept approach in a mouse osteotomy model. For the *in vitro* analysis, Iloprost was directly added to the osteogenic differentiation culture of bone marrow mesenchymal stromal cells (BM MSCs) isolated from the femur of C57BL/6N mice (Figure 1, left). To investigate an indirect effect of Iloprost on the mineralization capacity of osteogenic induced BM MSCs, all bone marrow cells or isolated CD8+ T cells from the bone marrow were stimulated with α-CD3/α-CD28 and the obtained conditioned media were added to the osteogenic differentiation culture of BM MSCs (Figure 1, right). The osteogenic differentiation was quantified based on mineralization by Alizarin Red staining.

### Cell Stimulation for the Production of Conditioned Media

Bone marrow and isolated CD8+ T cells were stimulated by an α-CD3/α-CD28 stimulation for 2 days in 96 well-plates. The stimulation was performed in RPMI 1640 media supplemented with 10% fetal bovine serum (FBS), 1% penicillin/streptomycin (P/S), 50 μM β-mercaptoethanol, and 10 ng/ml IL-2. In the respective experimental setup, either PBS or 300 nM or 3 μM Iloprost were added to the culture. 5 x 10^5^ cells in 225 μl were plated per well of a 96 well-plate. After the two stimulations, the supernatant was harvested (conditioned media) and stored at −80C.

### Isolation and Polarization of Macrophages

1 x 10^6^ isolated bone marrow cells were plated per well into a 96 well-plate and incubated for 3 days in RPMI complete media: RPMI 1640 supplemented with 50 ng/ml macrophage colony-stimulating factor (M-CSF), 1% P/S, 10% FBS, and 50 μM β-mercaptoethanol. Subsequently, RPMI complete media was replaced by the respective polarization media and cells were polarized for additional 3 days. For MΦ: RPMI complete media with PBS; M1: RPMI complete media with 20 ng/ml IFNγ and M2: 20 ng/ml IL-4/IL-13. The produced conditioned media was harvested and stored at −80°C. Macrophage monolayers were washed twice with PBS and fixed with 4% PFA/PBS for 10 min. Storage was done in PBS at 4°C for subsequent confirmation of polarization via immunofluorescence.

### Immunofluorescence Staining of Polarized Macrophage Subsets

The immunofluorescence staining of polarized macrophages were realized on fixed cellular macrophage monolayers. Cells were shortly washed with PBS, permeabilized in 100 μl PBS supplemented with 0.1% Tween for 30 min and subsequently blocked for 30 min with PBS supplemented with 5% FBS. The following antibodies were used for the staining: α-CD68 FITC, α-CD206 PE, and α-CD80 AF647. Antibodies were incubated for 1 h in the dark at RT. Cells were washed with PBS and cell nuclei were stained with DAPI for 10 min in the dark at RT. Cell monolayers were washed twice with PBS and wells were kept at 4°C in the dark until imaging. Imaging was performed with a standard fluorescence microscope (Axio Observer, Carl Zeiss).

### Investigation of the Immunomodulatory Effect of Iloprost

To investigate the immunomodulatory effect of Iloprost on immune cells, ELISA were performed analyzing the secretion of IFNγ, TNFα, and IL-10 as indicated. Frozen conditioned media were thawed and analyzed with respective ELISA kits following the manufacturer's protocol. ELISA was performed with a Mouse IFNγ ELISA Ready-SET-Go!®, Mouse TNFα ELISA Ready-SET-Go!®, and Mouse IL-10 ELISA Ready-SET-Go!® from eBioscience (Affymetrix, Santa Clara, CA USA). The samples were incubated at 4°C overnight. Final staining reactions were stopped with 1 M H_3_PO_4_ and absorbance values were acquired at 450 nm with a reference wavelength of 570 nm with Tecan Infinite M200 PRO (Tecan, Männedorf, Switzerland) and analyzed with i-control 1.9 software (Tecan, Männedorf, Switzerland).

### Isolation and Cultivation of Mesenchymal Stromal Cells

At least 3 x 10^7^ isolated bone marrow cells were cultured in expansion media: low glucose DMEM media supplemented with 10% FBS, 1% P/S, and 1% glutamax. Media exchange was performed twice a week to remove non-adherent cells until cultures were confluent. To detach the MSC monolayers, cells were washed once with PBS. TrypLE was added to the monolayer, incubated for 5 min at 37°C. The cell suspension was washed, centrifuged and the passaged cells were plated with increasing surface area.

### Osteogenic Differentiation of MSCs and Quantification by Alizarin Red Staining

1.5 x 10^4^ MSCs were seeded per well into a 96 well-plate. Cells were cultured in expansion media for 2 days. Subsequently, osteoinductive media was applied to the cells: low glucose DMEM media supplemented with 10% FBS, 100 nM dexamethasone, 10 mM β-glycerol phosphate, 50 μM L-ascorbate-2-phosphate, 1% P/S, and 1% glutamax. Osteogenic differentiation was stopped after 14 days including a media exchange every 3–4 days. When conditioned media of stimulated bone marrow or CD8+ T cells was supplemented, double concentrated osteoinductive media was mixed 1:2 with the respective conditioned media.

Alizarin Red staining was applied to quantify the osteogenic differentiation of the cultured MSCs. After a 14 days culture in osteoinductive media, well-plates were washed twice with PBS. Cell layers were fixed for 10 min at RT in 50 μl 4% paraformaldehyde/PBS (PFA/PBS). Cell nuclei were stained for DAPI for 10 min in the dark at RT. Cells were washed in ddH_2_O and incubated with 0.5% Alizarin Red for 10 min at RT. Cells were washed five times with ddH_2_O and cell layers were dried before imaging. For the quantification, Alizarin Red was detached with 10% cetylpyridinium chloride for 30 min at RT and optical density was measured and quantified with the plate reader Infinite M200 PRO (Tecan, Männedorf, Switzerland).

### Chondrogenic Differentiation of MSCs and Quantification by Histomorphometry

3 x 10^5^ MSCs were transferred into a 15 ml tube for the chondrogenic differentiation. Cells were centrifuged and chondrogenic induction media was carefully added to the cells without resuspension: high glucose DMEM media supplemented with 100 nM dexamethasone, 50 μg/ml L-ascorbate-2-phosphate, 350 μM L-proline, 2 mM sodium pyruvate, 6.25 μg/ml Insulin-transferrin-sodium selenite media supplement, 1.25 mg/ml bovine serum albumin, 5.35 μg/ml linoleic acid, 10 ng/ml TGF-β1, 10 ng/ml BMP-2, 1% P/S, and 1% glutamax. Pellets were cultured for 21 days under hypoxic conditions. Chondrogenic inductive media was changed twice a week.

For quantification, chondrogenic differentiated cell pellets were paraffin embedded, cut and stained with Alcian Blue (staining of the proteoglycans). Therefore, cell pellets were fixed for 2 h in 4% PFA/PBS, washed twice in PBS and dehydrated in an increasing alcohol series: 30 s in 70% EtOH, 20 min in 80% EtOH, 20 min 96% EtOH and twice 20 min in 100% EtOH. Cell pellets were incubated for 15 min in Xylol and subsequently paraffin embedded. Four micrometer thick sections were cut from three different areas of each pellet. Deparaffinized sections (incubation of the sections twice in Xylol for 10 min each and in a descending alcohol series) were washed in ddH_2_O for 2 min, equilibrated in 3% acetic acid for 3 min, stained in 1% Alcian Blue for 45 min, washed in 3% acetic acid, washed in ddH_2_O, and stained for cell nuclei in Nuclear Fast Red for 2 min. Pellets were shortly washed in ddH_2_O and 70% EtOH. After an ascending alcohol series, pellets were incubated twice in Xylol, 10 min each, and embedded. Acquired images were quantified based on the blue values of bright field images.

### Investigation of the Cellular Metabolic Activity by Prestoblue

Using the PrestoBlue Cell Viability Reagent (Thermo Fisher Scientific, Waltham, MA, USA), the metabolic activity of the stimulated cells was investigated after manufacturer's protocol. The reagent was diluted in RPMI complete media (for bone marrow cells) or low glucose DMEM (for MSCs), respectively, and applied to the cultured cells. After a 1 h incubation, the supernatant was collected and fluorescence top reading was performed at 560 nm excitation and 590 nm emission with the plate reader. For background correction, fluorescence values of no-cell control wells, which contained only reagent solution were averaged and subtracted from values of experimental wells.

### Setting of an Osteotomy for the *in vivo* Analysis

The pro-regenerative potential of Iloprost was evaluated in a mouse osteotomy model. Therefore, mice were anesthetised by inhalation of Isoflurane. Before surgery, the animals received subcutaneous injection of the analgesic Buprenorphine (0.03 mg/kg s.c.) and of the antibiotic Clindamycin (0.02 ml s.c.). The operation area of the left femur was shaved and disinfected. The skin was opened by a longitudinal cut from the knee to the hip. The femur was bluntly exposed and stabilized by an external fixator (MouseExFix, RISystem AG, Davos, Switzerland). An osteotomy of 0.7 mm was introduced between the middle pins using a Gigli wire saw (RISystem AG, Davos, Switzerland). A biphasic fibrin clot (loaded with either 3 μM Iloprost or PBS) (Tissucol-kit Immuno, Baxter) was inserted into the osteotomy gap. The skin was closed and sutured. Mice were brought back to the cage and observed until they were fully mobile again. As post-operative analgesia, Tramadol hydrochloride (0.1 mg/ml) was added to the drinking water for 3 days.

### Micro-Computed Tomography of Osteotomized Mouse Bones

To evaluate the healing outcome after Iloprost administration, fractured femora were harvested 21 days post-osteotomy and analyzed by μCT. Therefore, mice were euthanized by administering ketamine and xylazine (i.p., ketamine: 120 mg/kg, xylazine: 16 mg/kg) and cervical dislocation in deep anesthesia. After preparation of the femora, they were directly fixed in 4% PFA/PBS for 4 h at 4°C. Subsequently, the bones were dehydrated in an ascending sugar series: 10, 20, and 30%, for 24 h for each at 4°C. The bones were scanned in a μCT Viva 40 (SCANCO Medical AG, Brüttisellen, Switzerland). For the scan, the following parameters were used: 10.5 μm voxel size, 55keVp, and 145 uA. A gray value threshold was defined before analysis in order to be able to distinguish between mineralized and non-mineralized bone tissue (27). The global threshold for defining mineralized bone was set to 242, which corresponds to a mineralization of 369.9 mg hydroxyapatite (HA)/cm^2^. The scanned volume of interest (VOI) included 190 slices around the middle of the fracture gap to the distal and proximal part of the femur, respectively. For the quantification of the μCT data, cortical bone was excluded from newly formed mineralized bone.

### Histological and Immunohistological Analysis

For histological and immunohistological analysis, fractured bones were cryo embedded. Seven micrometer thick sections were cut and stained either for Movat's Pentachrome (overview staining) or the following cell types (immunofluorescence staining): CD4+ and CD8+ T cells, IFNγ-producing CD8+ T cells, osteoblasts, osteoclasts and differentially polarized macrophages.

The Movat pentachrome staining was done as follows: cryo-sections were thawed for 1 h at RT and fixed for 10 min in 4% PFA/PBS. Sections were washed twice in PBS/Tween-20 for 5 min. Subsequently, sections were incubated for 3 min in 3% acetic acid, for 30 min in 1% Alcian Blue/3% acetic acid, and differentiated for 5 min in 3% acetic acid. After washing in ddH_2_O, sections were incubated for 1 h in ethyl alcohol, washed twice in tap water, shortly in ddH_2_O and stained in iron hematoxylin (after Weigert) for 10 min. After washing with tap water, sections were incubated for 15 min in brilliant crocein-acid fuchsine. Tissue slides was shortly placed in 0.5% acidic acid, followed by a 20 min incubation in 5% phosphotungstic acid. After 1 min in 0.5% acetic acid, slides were incubated 3x à 5 min in 96% EtOH and stained with Saffron-du-Gatinais for 1 h. Subsequently, they were washed again 3x in 96% EtOH for 2 min each, 2x in Xylol for 10 min each and embedded.

Analysis of the immune cell subsets and osteoblasts, osteoclasts was done by immunofluorescence staining. All steps were performed at RT in a humidified chamber. Thawed cryo-sections were fixed for 20 min in 4% PFA/PBS/Tween-20 and washed twice with PBS/Tween-20. Sections were blocked in 1x TBS supplemented with 7% FBS and 0.05% Tween-20 for 1 h. Blocking buffer was decanted and the primary antibodies were applied to the respective section in the following combinations: (1) CD4+ and CD8+ T cells and osteoblasts: CD4 AF594, CD8 PE, osteocalcin; (2) IFNγ-producing CD8+ T cells: CD8 PE and IFNγ; and (3) differentially polarized macrophages: CD68 FITC, CD206 PE, and CD80 AF647. Tissue sections were washed in 1x TBS and, if necessary, incubated for 2 h with a secondary antibody: anti-rabbit AF647 for osteocalcin or anti-rat AF594 for IFNγ and for CD4. For the staining of osteoclasts, antigen retrieval was performed with ProteinaseK for 15 min on thawed cryo-sections. Sections were washed PBS/Tween-20 and fixed as described above. After washing and blocking, sections were stained for cathepsinK in 3.5% FBS, 0.025% Tween in tris-buffered saline (TBS) for 2 h. Sections were washed and stained for CD68 (FITC) and the secondary antibody for cathepsinK anti-rabbit AF647 in 3.5% FBS, 0.025% Tween in TBS for 2 h. Sections were washed, stained for cell nuclei with DAPI for 10 min and subsequently embedded. Sections were analyzed with a laser scanning microscope LSM 710 (Carl Zeiss AG, Oberkochen, Germany).

### Statistics

The statistical evaluation of the presented data was done with the programs Graph Pad Prism and SPSS. Data were presented as dot plot graphs. Statistics were done by using the Mann-Whitney U test and data were statistically significant if *p* ≤ 0.05. For comparison of more than two study groups the Bonferroni's *post-hoc* test was used.

## Results

### Immunomodulatory Effects of Iloprost on Immune Cells

We first tested the immunomodulatory properties of Iloprost on murine immune and mesenchymal stromal cells (MSCs) *in vitro*. Both, immune cells and MSCs are known to be essential for the early healing phase in bone regeneration.

**Figure 2:**
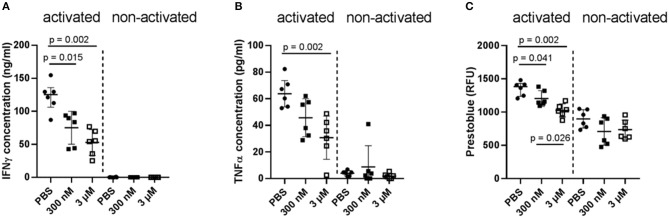
Immunomodulatory effects of Iloprost on bone marrow cells. Bone marrow cells were stimulated for 2 days by α-CD3/α-CD28 in addition to either PBS or two different concentrations of Iloprost (300 nM or 3 μM, respectively). The following secreted cytokine concentrations were evaluated: IFNγ **(A)** and TNFα **(B)**. The metabolic activity of the stimulated bone marrow cells was measured via Prestoblue **(C)**. *n* = 6.

In a first attempt, two different concentrations of Iloprost were tested on the whole bone marrow cellular composition: 300 nM and 3 μM. As readout, the secretion of the pro-inflammatory cytokines IFNγ and TNFα was analyzed. Both cytokines play an important role as signaling molecules in bone repair, especially in the early fracture healing phase. However, too high amounts of them negatively affect bone repair by diminishing the formation of mineralized matrix by MSCs (15). After a 2-day stimulation of the cells by the different concentrations of Iloprost, the concentration of secreted IFNγ and TNFα was significantly decreased in comparison to the control (PBS supplementation) (IFNγ: ~130 to ~55 ng/ml; TNFα: ~65 to ~40 pg/ml) (Figures 2A,B). Comparing the two different concentrations of Iloprost, the supplementation of the higher one (3 μM) led to an even more pronounced decrease of the secreted cytokines. As expected, non-activated cells showed almost no secretion of IFNγ and TNFα. The metabolic activity of the stimulated bone marrow cells was also downregulated by the supplementation of Iloprost in comparison to the control (Figure 2C).

**Figure 3:**
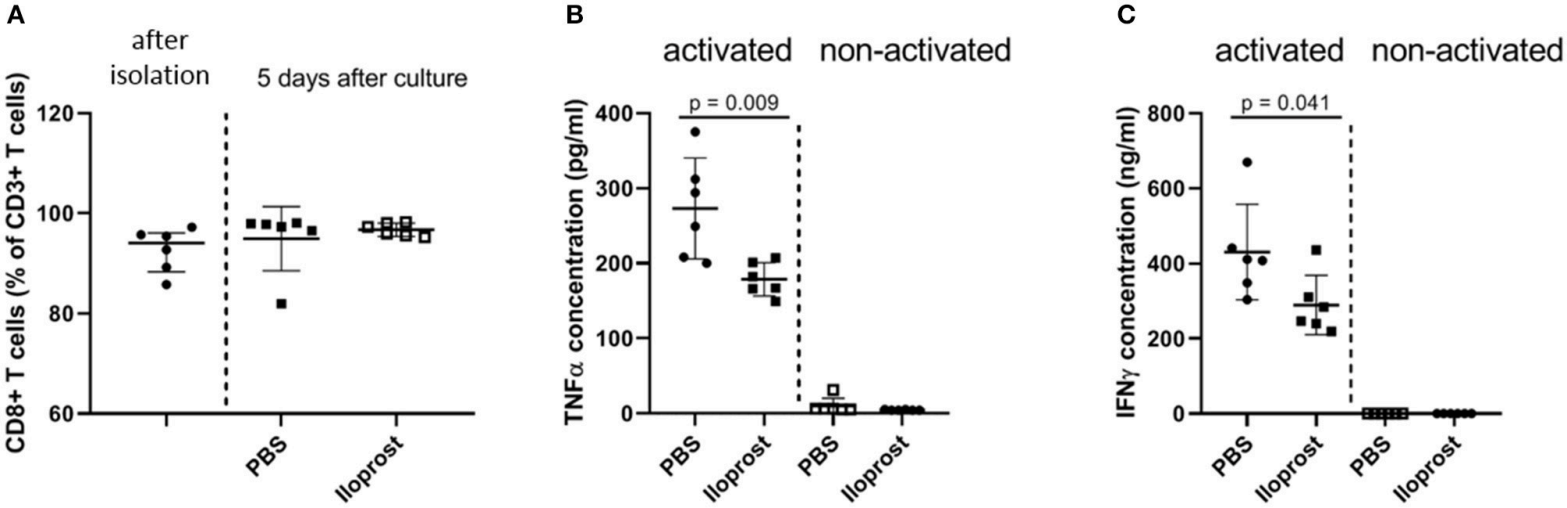
Immunomodulatory effects of Iloprost on isolated CD8+ T cells. The purity of the isolated CD8+ T cells after bead-based separation as well as after *in vitro* stimulation was confirmed by flow cytometry **(A)**. CD8+ T cells were stimulated by α-CD3/α-CD28 in addition to either PBS or 3 μM Iloprost. The following secreted cytokine concentrations were evaluated: TNFα **(B)** and IFNγ **(C)**; *n* = 6.

In patients, we already showed that a too high amount of CD8+ T cells, one special subset of the adaptive immunity, negatively regulates successful bone repair (15). CD8+ T cells are one of the main producer of pro-inflammatory cytokines in the early bone repair phase. Therefore, as a next step, we evaluated the immunomodulatory effect of Iloprost on murine CD8+ T cells. CD8+ T cells were isolated via PluriBeads (pluriSelect) from bone marrow and spleen. The purity of the isolated cells was confirmed by flow cytometry after separation as well as after the duration of the *in vitro* stimulation and stayed above 80% (Figure 3A). Similar to the stimulation of the whole bone marrow cellular fraction, isolated CD8+ T cells showed a decreased secretion of IFNγ and TNFα under the presence of 3 μM Iloprost in comparison to the control (IFNγ: ~410 to ~250 ng/ml; TNFα: ~275 to ~180 pg/ml) (Figures 3B,C). The metabolic activity was again slightly downregulated by the Iloprost supplementation (Figure S1).

**Figure 4:**
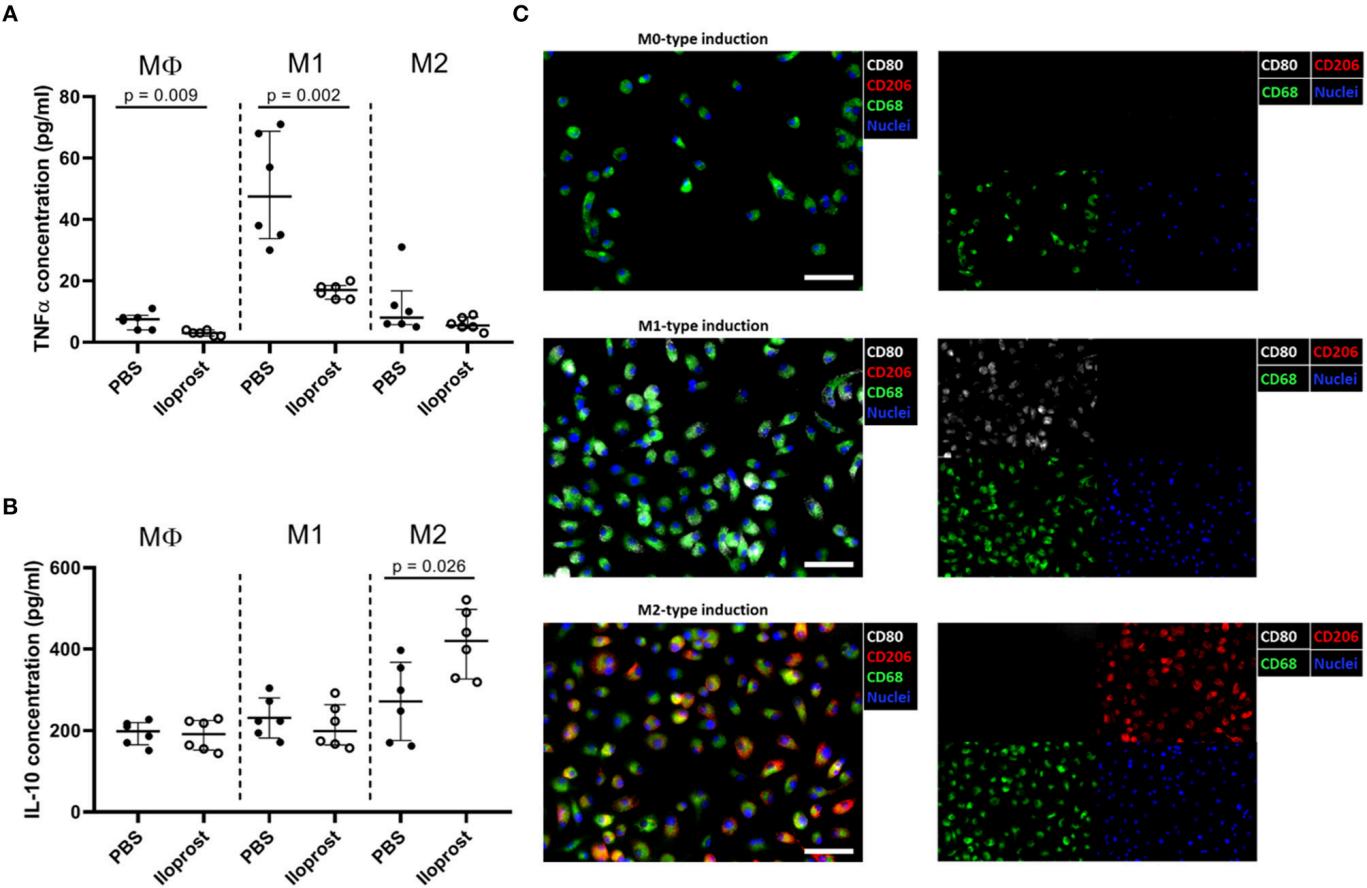
Immunomodulatory effects of Iloprost on polarized bone marrow macrophages. Macrophages were polarized for 3 days toward the M1 or M2 type, supplemented with either PBS or 3 μM Iloprost. **(A,B)** The following secreted cytokine concentrations were evaluated: TNFα **(A)** and IL-10 **(B)** of M1-type induced and M2-type induced polarized macrophages, respectively. **(C)** Representative immune fluorescence images of unpolarised CD68+ MΦ macrophages (on top), CD68+CD80+ M1 macrophages (middle), and CD68+CD206+ M2 macrophages (bottom). Color code of the immune fluorescence images: CD68 = green, CD80 = white, CD206 = red, and cell nuclei = blue (DAPI). Scale bars: 100 μm; *n* = 6.

Besides cells of the adaptive immunity, also cellular compartments of the innate immune system play a key role in the early fracture healing phase. Macrophages are one of the first cells infiltrating the fracture area and are necessary for (a) the clearance of the cell debris as well as for (b) the recruitment of further cells important for the progression of the healing cascade due to their secreted cytokine profile (16, 28). We already demonstrated the importance of macrophages in bone regeneration using an *in vivo* mouse osteotomy model. After a chemically induced reduction of the macrophage cell population, a disturbed bone healing outcome was observed in comparison to the control group while an induction of the regulatory M2 macrophage phenotype (addition of IL-4/IL-13) lead to a significantly enhanced healing outcome (16). In the here presented study, we further tested whether the supplementation of Iloprost promotes simultaneously the downregulation of pro-inflammatory and the upregulation of anti-inflammatory cytokines by MΦ, M1, or M2 polarized macrophages, respectively (Figure 4). Regarding the secretion of TNFα, the supplementation of Iloprost led to a decreased secretion by MΦ as well as by pro-inflammatory M1 (~50 to ~20 pg/ml) (Figure 4A). Whereas, the secretion of the anti-inflammatory cytokine IL-10 was significantly upregulated in the M2 type, but unaffected in the MΦ and M1 macrophages (~290 to ~410 pg/ml) (Figure 4B). The polarization culture was confirmed by immune fluorescence staining of the stimulated and polarized cells (Figure 4C). MΦ macrophages were identified by the marker expression CD68 (green fluorescence signal). M1 were double positive for CD68 and CD80 (CD80: white fluorescence signal) and M2 double positive for CD68 and CD206 (CD206: red fluorescence signal). Cell nuclei were identified by DAPI (blue fluorescence signal).

### Effects of Iloprost on the Osteogenic and Chondrogenic Differentiation Capacity of Mesenchymal Stromal Cells

MSCs are the precursor cells for cartilage producing chondrocytes and bone forming osteoblasts. During secondary bone healing, a cartilage template is first build. These chondrocytes further get hypertrophic, mineralize and are subsequently replaced by newly formed woven bone produced by osteoblasts. Thus, a cartilage template is indispensable for successful bone regeneration. Therefore, the osteogenic and chondrogenic differentiation capacity of MSCs was investigated under the influence of Iloprost. We tested again two different concentrations of Iloprost: 300 nM and 3 μM, respectively.

**Figure 5:**
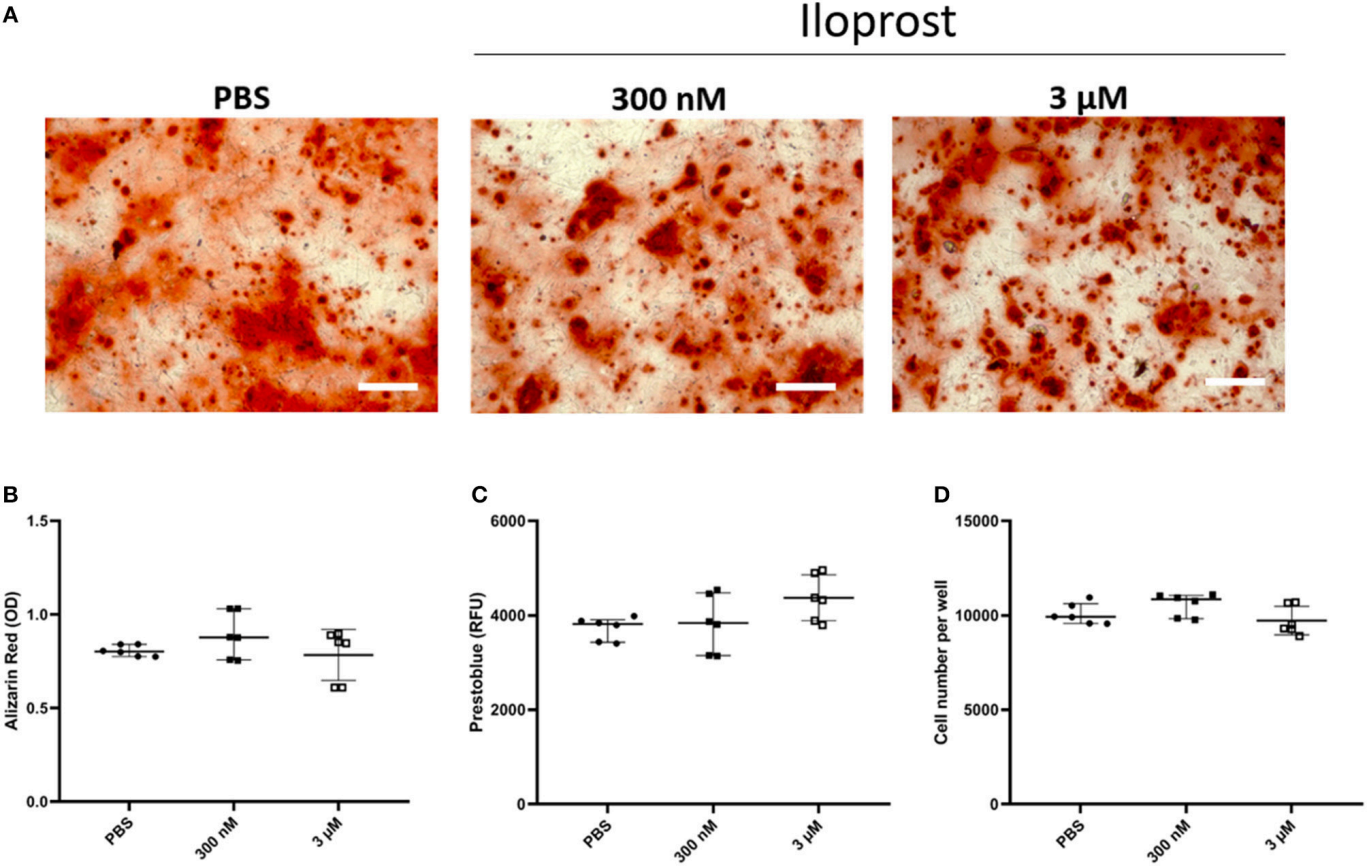
Effect of Iloprost on mineralization capacity of osteogenically induced MSCs. Monolayers of MSCs were stained with Alizarin Red to visualize the formation of mineralized matrix. **(A,B)** Representative images of the Alizarin Red staining of cultured MSCs with osteoinductive media after 14 days of cultivation. Different concentrations of Iloprost were supplemented to the osteoinductive media (300 nM and 3 μM, respectively) **(A)**. Quantification of the Alizarin Red staining **(B)**. The metabolic activity of the cultured MSCs was measured by Prestoblue **(C)**. Determination of the cell number of the cultured MSCs under the different stimuli **(D)**. Scale bars: 200 μm; *n* = 6.

For the osteogenic differentiation, monolayers of MSCs cultured for 14 days in osteoinductive media were stained with Alizarin Red to reveal the calcification of the cells (Figure 5). The quantification of the Alizarin Red staining demonstrated that Iloprost had no negative effect on the osteogenic capacity of MSCs (Figures 5A,B). The metabolic activity as well as the cell number of the cultured MSCs were also unaffected by the presence of Iloprost in the osteoinductive media (Figures 5C,D).

**Figure 6:**
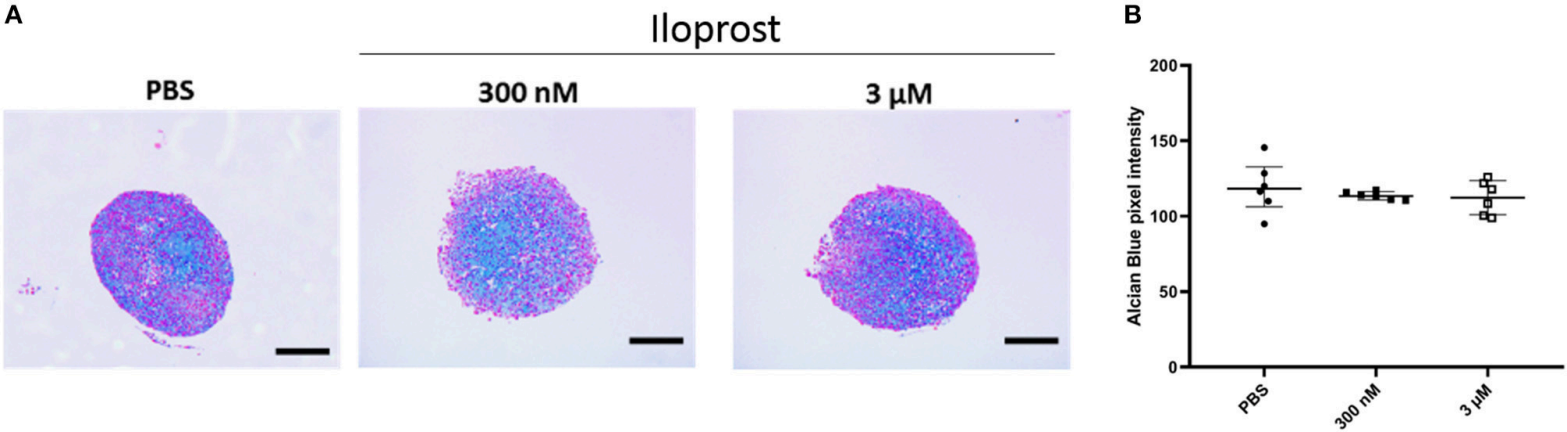
Effect of Iloprost on cartilage formation capacity of chondrogenically induced MSCs. **(A)** Alcian Blue stained paraffin sections of cartilage pellets after 21 days of incubation in chondroinductive media, supplemented with different concentration of Iloprost (300 nM and 3 μM, respectively) or PBS as control. **(B)** Quantification of the Alcian Blue staining. Scale bars: 200 μm; *n* = 6.

After the demonstration that Iloprost is not affecting the mineralization capacity of MSCs, we evaluated the impact of Iloprost on their capacity to differentiate into the chondrogenic cell lineage. Representative images of Alcian Blue stained paraffin sections of cartilage pellets are presented in Figure 6A. The quantification of the Alcian Blue staining revealed no negative effect on the proteoglycan production of chondrogenically induced MSC pellets under the supplementation of Iloprost in comparison to the PBS control (Figure 6B).

### Osteoimmunological Effect of Iloprost

In the first part of the study, we demonstrated that the supplementation of Iloprost promotes the functionality of immune cells toward both, a reduction of pro-inflammatory signals and an induction of anti-inflammatory ones. We further showed that Iloprost had no negative effect on the osteogenic as well as chondrogenic differentiation capacity of MSCs. Next, we wondered, whether the immune modulatory effect of Iloprost on immune cells is also influencing the osteogenic capacity of MSCs.

**Figure 7:**
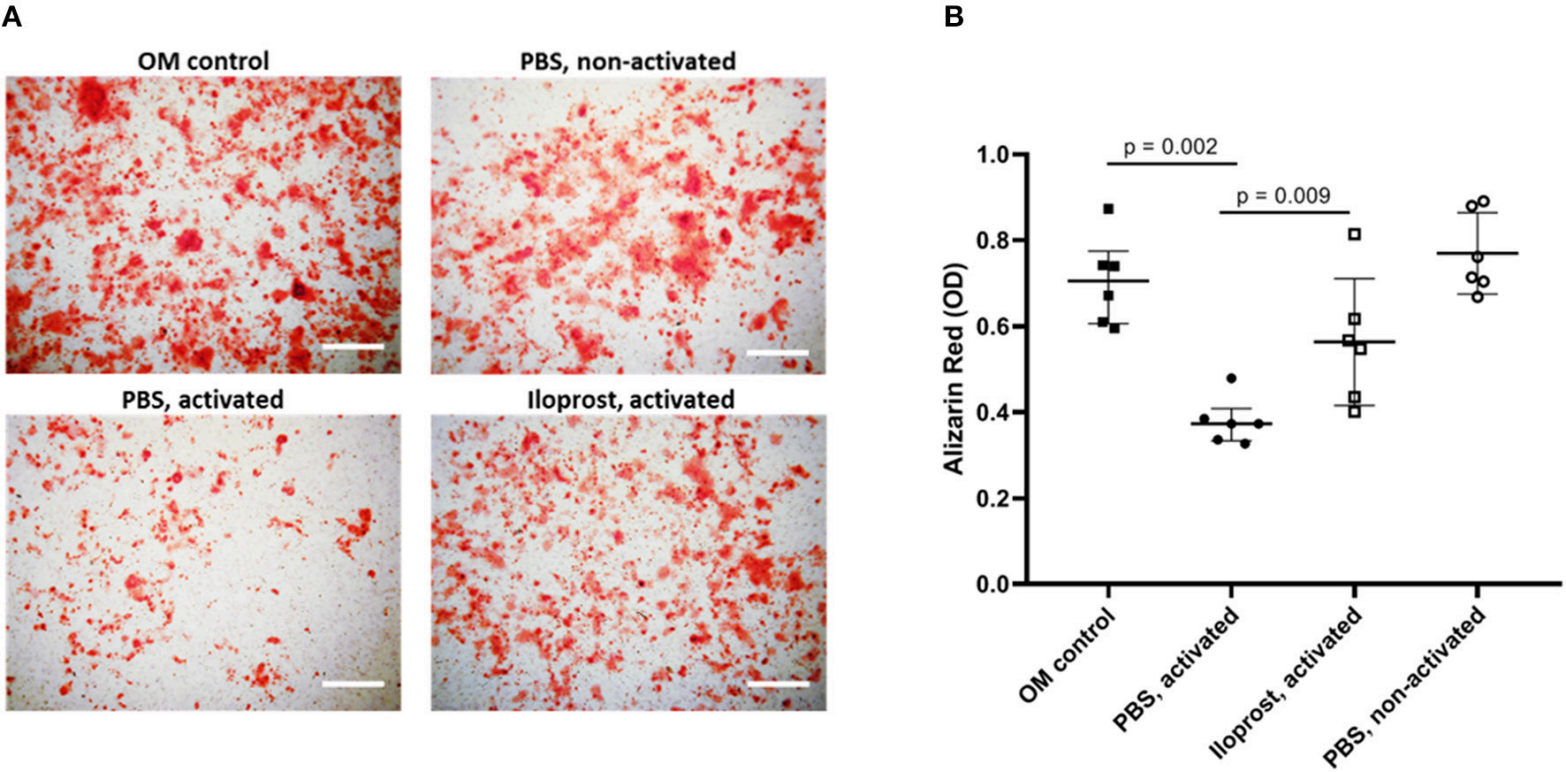
Osteoimmunological effect of Iloprost treated bone marrow cells. α-CD3/α-CD28 activated bone marrow cells were cultured either with Iloprost or PBS as control. Obtained conditioned media was added to an osteogenic differentiation culture of MSCs and the secretion of mineralized matrix was measured by Alizarin Red staining. **(A)** Representative images of the Alizarin Red staining of the different culture conditions. **(B)** Quantification of the Alizarin Red signal. OM, osteoinductive media. Scale bars: 200 μm; *n* = 6.

Therefore, conditioned media (CM) of α-CD3/α-CD28 stimulated and Iloprost treated bone marrow cells and isolated CD8+ T cells, respectively, were added to the osteoinductive culture of MSCs. The CM of activated bone marrow cells significantly decreased the mineralization capacity of MSCs in comparison to the control (cultivated MSCs in osteoinductive media, OM) (Figures 7A,B). The supplementation of Iloprost during the α-CD3/α-CD28 stimulation of bone marrow cells was able to compensate partially the negative impact on mineralization induced by the activation (Iloprost, activated). CM of non-activated bone marrow cells had no effect on the mineralization capacity of MSCs (PBS, non-activated).

**Figure 8:**
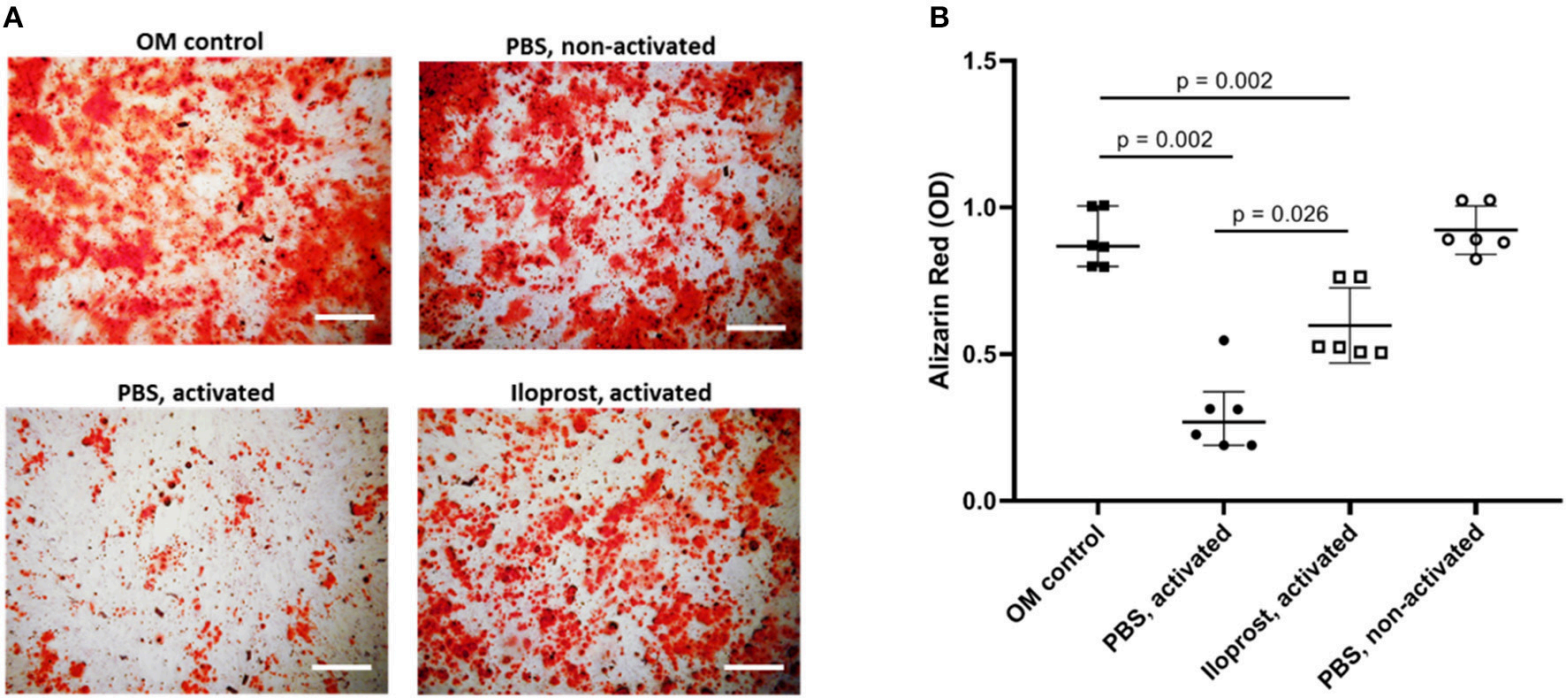
Osteoimmunological effect of Iloprost treated isolated CD8+ T cells. Isolated α-CD3/α-CD28 activate CD8+ T cells were cultured either with Iloprost or PBS as control. Obtained conditioned media was added to an osteogenic differentiation culture of MSCs and the secretion of mineralized matrix was measured by Alizarin Red staining. **(A)** Representative images of the Alizarin Red staining of the different culture conditions. **(B)** Quantification of the Alizarin Red signal. OM, osteoinductive media. Scale bars: 200 μm; *n* = 6.

Repeating the analysis of the osteoimmunological effect of Iloprost on MSCs with isolated CD8+ T cells, similar results were obtained. CM of activated CD8+ T cells led to a significant decrease of mineralized matrix synthesis of cultured MSCs compared to the control (MSCs cultured in OM) (Figures 8A,B). Again, the supplementation of Iloprost to the stimulation of CD8+ T cells significantly improved the osteogenic matrix production by MSCs (Figures 8A,B; Iloprost, activated). However, this was still significantly lower in comparison to the OM control. CM of non-activated CD8+ T cells had no effect on the mineralization of MSCs (PBS, non-activated).

Summarizing the data from our *in vitro* study, we confirmed the immune modulatory effect of Iloprost on the secreted cytokine profile of immune cells. We also showed that Iloprost had no effect on the cartilage and bone forming capacity of MSCs. Thus, Iloprost is not negatively affecting the pro-regenerative functionality of MSCs. In a next step, we tested the capacity of Iloprost as a bone healing promoting agent in our mouse osteotomy model in a proof of concept *in vivo* approach.

### Iloprost–A Potent Agent to Promote Bone Fracture Healing *in vivo*?

In bone regeneration, a first pro-inflammatory phase is indispensable for the initiation of the healing cascade. Due to the anti-inflammatory effect of Iloprost via its effect on immune cell function shown *in vitro*, we chose an application strategy, where the applied Iloprost will be successively released from a biphasic fibrin scaffold, thus allowing the pro-inflammatory phase to proceed. Fibrous tissue is a component of the classical healing cascade in bone regeneration and thus represents an endogenous material, which is already present in the fracture gap. Furthermore, fibrin is biocompatible and biodegradable. Iloprost embedded in a fibrin clot was inserted during surgery in the osteotomy gap. Due to the biphasic structure of the fibrin scaffold, the included Iloprost would not be directly released in the fracture zone at the onset of the surgery but with a delay. This delay allows the initial pro-inflammatory phase to proceed and to initiate the healing cascade.

**Figure 9:**
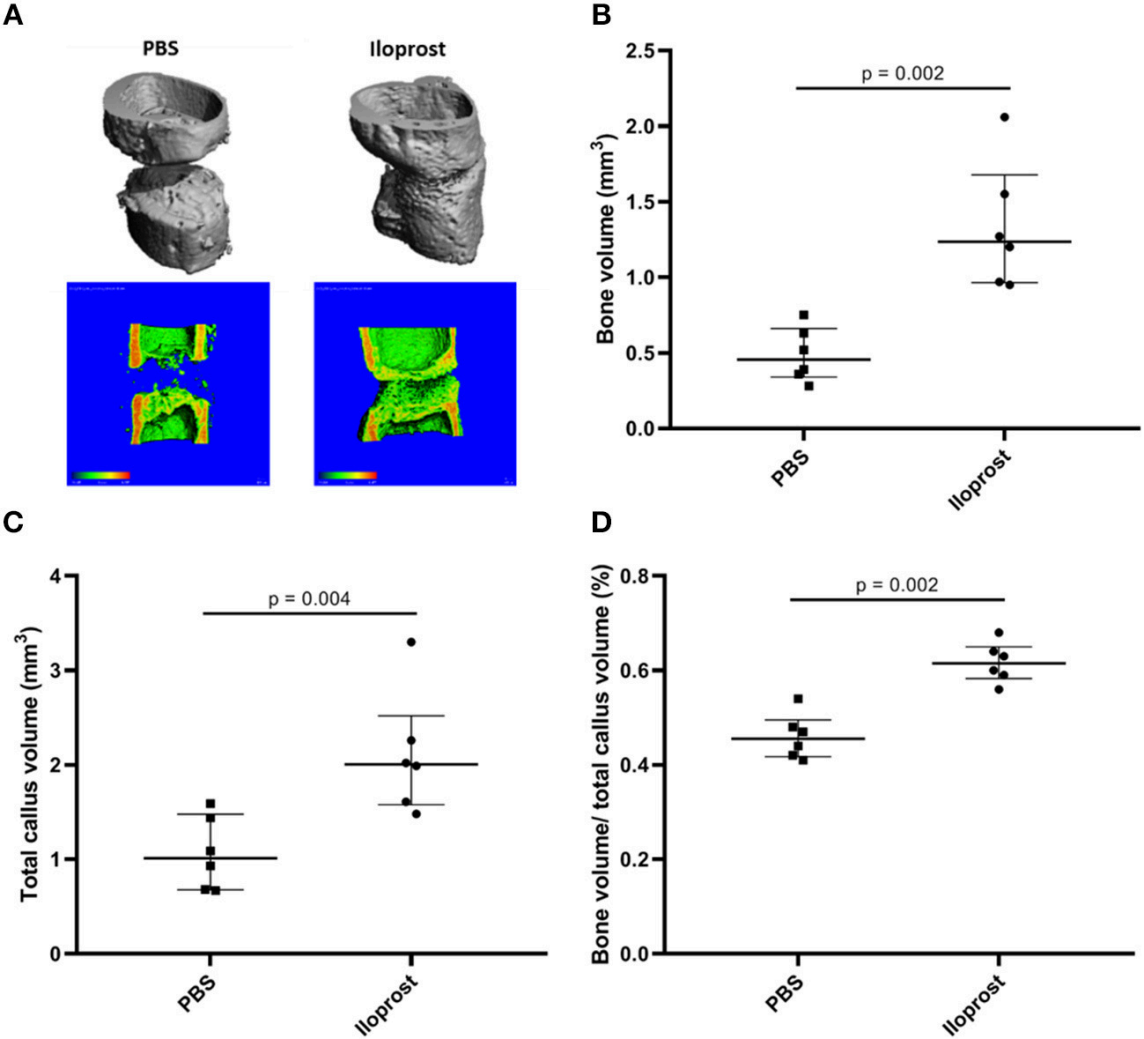
Proof of concept approach of Iloprost to improve bone fracture healing *in vivo*. μCT analysis of the healing outcome 21 days post-osteotomy in a mouse osteotomy model. **(A)** Representative μCT images of the Iloprost treated (right) and control (left, PBS) group. The upper row shows the 3D reconstruction of the μCT measurement. In the lower row, 3D reconstruction was cut in the middle to evaluate the healing progression. The color code indicates the mineralization state, increasing from blue to red. **(B–D)** Quantification of the μCT results; *n* = 6.

The healing outcome was evaluated 21 days post-surgery. The model was chosen to enable detection of healing enhancement–with a gap size of 0.7 mm the healing is not concluded after 21 days (control) (Figure 9, PBS). Regarding the Iloprost treated group, μCT analysis 21 days post-surgery showed an improved healing outcome of the mice receiving Iloprost in comparison to the control group (mice with fibrin scaffold, PBS supplementation) (Figure 9; Iloprost: mice with Iloprost supplementation; PBS: control group). The quantification of the μCT data confirmed the already visually seen improved healing by a significant increase of bone volume, total callus volume and the ratio of bone volume/total callus volume in the Iloprost treated animals with regard to the control (Figures 9B–D).

**Figure 10:**
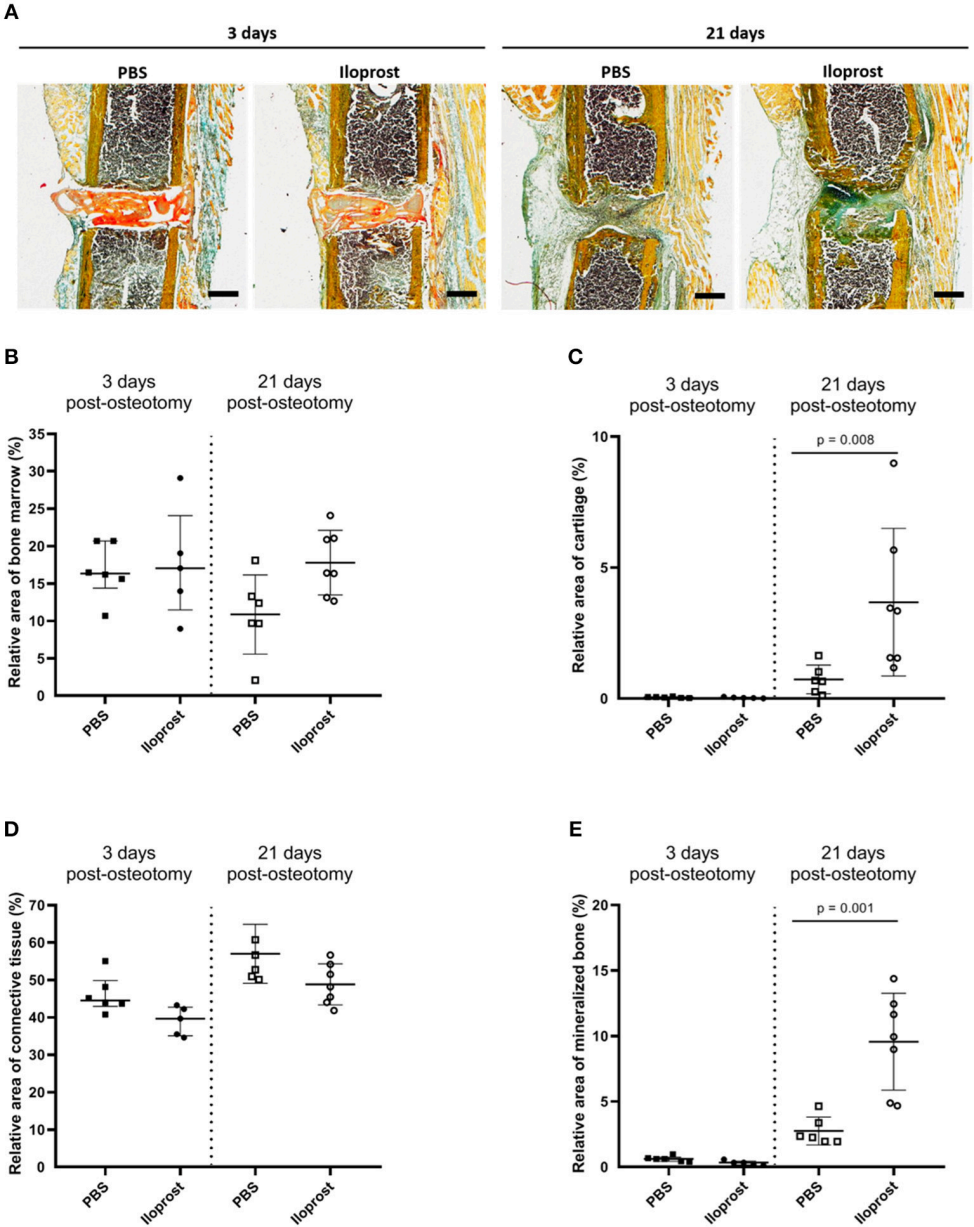
Histological analysis of *in vivo* experiments in mouse osteotomies at days 3 and 21 post-osteotomy. **(A)** Representative Movat's pentachrome pictures are displayed for the respective time point and group. **(B–E)** Histomorphometrical analysis of the Movat Pentachrome stainings: **(B)** bone marrow, **(C)** cartilage tissue, **(D)** connective tissue, and **(E)** mineralized bone tissue. Color coding of the Movat Pentachrome staining: mineralized bone = yellow, cartilage = dark blue-green, connective tissue = light blue-green, muscle fibers = light orange, cell nuclei = purple, fibrin clot = red/orange. Scale bars equal 500 μm, *n* = 6.

**Figure 11:**
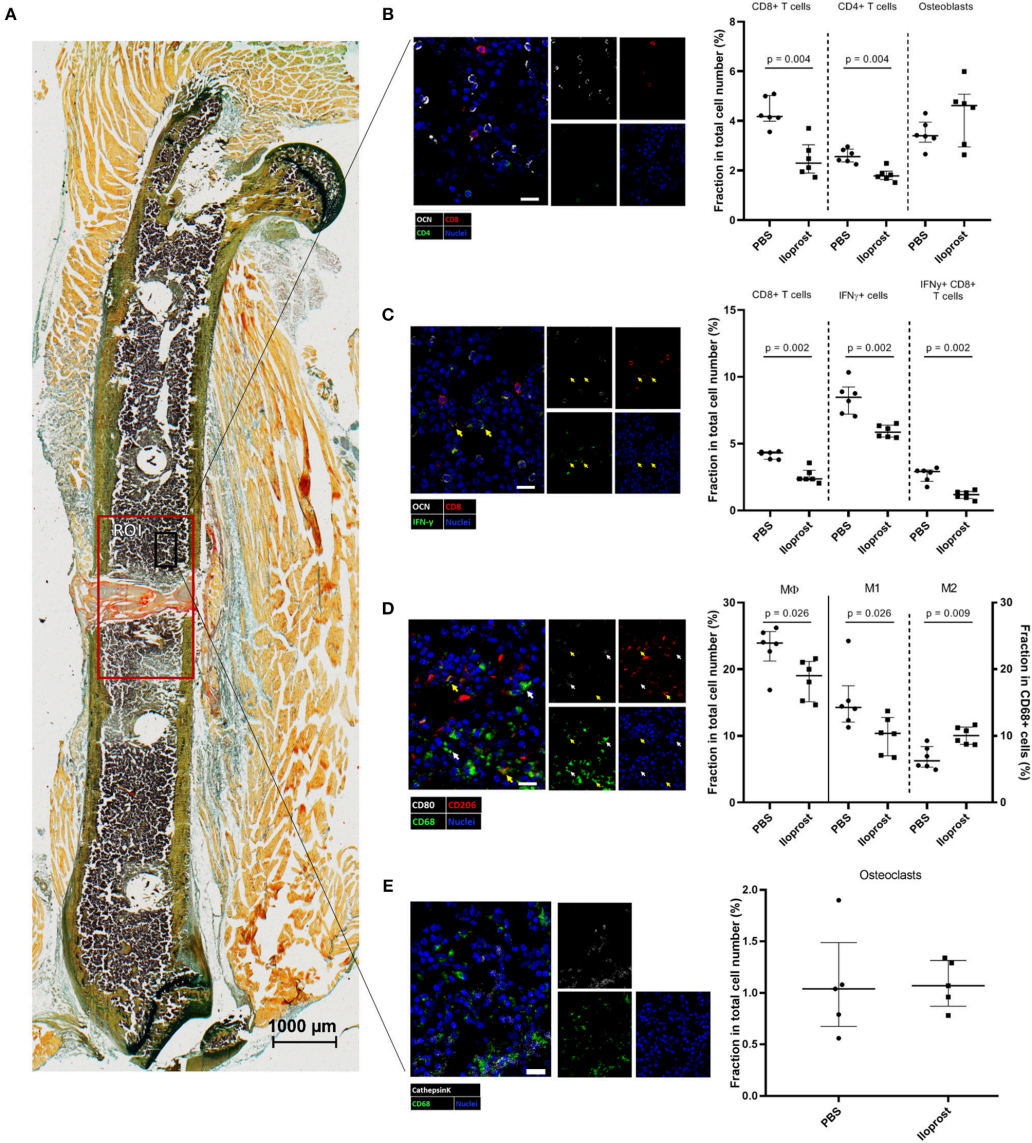
Immunohistochemical (IHC) analysis of the distribution of immune cells in the fracture zone 3 days post-osteotomy. **(A)** A section of a fractured femur from the Iloprost treated group is displayed, Movat Pentachrome staining. The region of interest (ROI) for the quantitative analysis of IHC images is enlarged (red rectangle). The black rectangle represents the region from which the representative examples of the respective IHC staining for **(B)** CD4+ and CD8+ T cells and osteoblasts, **(C)** IFNγ producing CD8+ T cells; CD8+IFNγ+ cells = white indicator, **(D)** macrophages; M1-type macrophages = white indicator; M2-type macrophages = yellow indicator, and **(E)** osteoclasts were taken. The quantification of the cellular distributions are displayed in the corresponding dot plot graphs. Color code: Movat Pentachrome mineralized bone = yellow, cartilage = dark blue-green, connective tissue = light blue-green, muscle fibers = light orange, cell nuclei = purple, fibrin clot = red/orange; **(B)** osteoblasts = white, CD8+ T cells = red, CD4+ T cells = green and cell nuclei = blue; **(C)** osteoblasts = white, CD8+ T cells = red, IFNγ = green, cell nuclei = blue; **(D)** CD68 = green, CD80 = white, CD206 = red; **(E)** osteoclasts: double positive for cathepsinK = white and CD68 = green cell nuclei = blue. Scale bars: 20 μm (IHC) 1,000 μm (Movat Pentachrome); *n* = 6.

Next to the μCT evaluation, histological and immunohistological analyses were performed on cryosections of the fractured femora 3 days and 21 days post-osteotomy (Figures 10, 11). Movat Pentachrome staining was performed to evaluate the relative amount of mineralized bone, cartilage, connective tissue and bone marrow (Figure 10). In Figure 10A, representative Movat Pentachrome pictures are presented to illustrate the tissue formation in and around the osteotomy gap at the respective time point. Three days post-surgery, the fibrin scaffold was still visible within the osteotomy gap in both groups, the control (PBS) and Iloprost treated animals (Iloprost), indicated by a red color after Movat's Pentachrome staining (Figure 10A, left). After 21 days, no fibrin scaffold was detected anymore in or around the osteotomy area (Figure 10A, right). Histomorphometrical analysis of the tissue distribution revealed a significantly higher amount of mineralized bone and cartilage tissue 21 days post-surgery in the Iloprost treated group in comparison to the control animals (Figure 10B). Three days post-osteotomy, both groups showed almost no proportion of mineralized bone or cartilage tissue. Both groups showed just slightly differences in the amount of connective tissue at both investigated time points (Figure 10D, connective tissue). Regarding the area of bone marrow, the Iloprost treated mice displayed a slightly higher amount 21 days after surgery.

To better understand the impact and direct influence of Iloprost during the early phase of bone healing, immunohistochemical (IHC) analyses were performed on bone sections of mice sacrificed 3 days post-osteotomy. At this time point, Iloprost conducted its immune modulatory effect as shown below (Figure 9). In addition, around 3 days post-osteotomy, the starting shift of the pro-inflammatory into the anti-inflammatory phase is observed in our chosen mouse osteotomy model system.

First, the distribution of CD4+ and CD8+ T cells (identified by CD4 and CD8, respectively), as well as osteoblasts (identified by osteocalcin, OCN) was investigated in a defined region of interest (ROI) in and around the fracture zone (Figures 11A,B). Quantification of the analyzed bone sections revealed a significantly reduction of the relative amount of CD4+ and CD8+ T cells in the Iloprost treated mice in comparison to the control. The relative number of osteoblasts was not affected by Iloprost treatment. Next, we were interested in the IFNγ producing CD8+ T cells, which were already shown to be detrimental for successful bone repair. Iloprost treatment led to a clear decrease of IFNγ producing CD8+ T cells with regard to the control group (Figure 11C, highlighted by arrows). Next to the T cells, the distribution of MΦ, M1, and M2 type macrophages was also evaluated 3 days post-osteotomy. Corresponding to the chosen marker set for the *in vitro* analysis, CD68 was used as pan-macrophage marker, CD68 and CD80 for M1 and CD68 and CD206 for M2 (Figure 11D). Due to the Iloprost administration in the fracture zone, a simultaneous significant decrease of pro-inflammatory M1 and significant increase of anti-inflammatory and pro-regenerative M2 macrophages was detectable. In order to evaluate if Iloprost administration has an impact on bone resorption, osteoclasts were also investigated by IHC. Osteoclasts were identified by the myeloid-lineage marker CD68, the collagen digesting enzyme cathepsinK (CTSK) and presence of multiple nuclei (Figure 11E). No significant differences were observed in both groups 3 days post-osteotomy.

Summarizing the data from the proof of concept study in our *in vivo* mouse osteotomy model system, the pro-regenerative effect of Iloprost in bone healing was confirmed. We further revealed underlying changes in the immune cell composition in and around the fracture zone in the early inflammatory phase toward a reduced pro-inflammatory and increased anti-inflammatory cell phenotype caused by the application of Iloprost. Thus, Iloprost is a promising agent to improve bone regeneration by the downregulation of partial unfavorable pro-inflammatory and a simultaneous support of anti-inflammatory and pro-regenerative mediators.

## Discussion

Healing is impaired if a prolonged (pro-) inflammatory phase persists and an early anti-inflammatory stimulus is required. Thus, developing strategies that would ensure or even enhance anti-inflammatory stimuli appears mandatory. Iloprost is a well-known drug to treat diseases of the vascular system like pulmonary arterial hypertension and scleroderma (25, 29, 30). Its main function is conducted via vasodilatation (widening of blood vessels). Regarding the bone system, Iloprost is already successfully used to treat bone marrow oedema, partially also in the context of bone injuries (23, 24).

In the here presented study, the immune modulatory effect of the synthetic prostacyclin analog Iloprost was evaluated for bone fracture healing. The impact of Iloprost was investigated on immune cells and MSCs *in vitro* as well as in a well-established mouse osteotomy model *in vivo*. We demonstrated that the supplementation of Iloprost led to a decrease in the expression of pro-inflammatory cytokines by immune cells (T cells and macrophages) and thus promoted a shift in the function of these cells toward the anti-inflammatory path. The metabolic activity of the stimulated immune cells was also downregulated by Iloprost further supporting the observed decrease of the secreted pro-inflammatory cytokine profile. Iloprost had no negative effect on the osteogenic as well as chondrogenic function of MSCs. In an *in vivo* proof of concept approach, the pro-regenerative capacity of Iloprost as potential agent to further bone regeneration was confirmed, evaluated by an improved healing outcome after 21 days. Immunohistochemical analysis of the cellular distribution in the fracture gap revealed a decrease of potential unfavorable IFNγ producing CD8+ cells as well as an increase of anti-inflammatory M2 macrophages in comparison to the control group due to Iloprost administration concurring with the *in vitro* results. Both are furthering the pro-regenerative pathway.

The signaling receptor for prostacyclin and thus also for Iloprost is found on a variety of different cell types, among others on cells of the innate as well as adaptive immunity. Iloprost signaling leads to an intracellular increase of cyclic adenosine monophosphate (cAMP) via the stimulation of the adenylyl cyclase. cAMP is an anti-inflammatory acting agent that suppresses the effector function of CD4+ and CD8+ T cells (31, 32). Here, we also observed a decrease of the secretion of the pro-inflammatory cytokines TNFα and IFNγ from whole bone marrow cells as well as (IFNγ producing) CD8+ T cells *in vitro* and *in vivo* under the presence of Iloprost. This observation demonstrates the immune modulatory effect of Iloprost on cellular components of the adaptive immunity toward a reduced pro-inflammatory and thus pro-regenerative phenotype. This finding was further confirmed by the increase of the pro-regenerative M2 type macrophages under the influence of Iloprost. The group of Alkhabit also showed an immune modulatory effect of Iloprost on macrophage polarization toward a pro-regenerative phenotype in an *in vivo* model system in rats, supporting our findings (33). Regarding bone fracture healing, possible negative effects of Iloprost on MSCs and bone forming osteoblasts as well as bone resorbing osteoclasts have to be considered. No possible negative effects by Iloprost on the osteogenic and chondrogenic capacities of MSCs were observed *in vitro*, further supporting the local application of Iloprost for bone healing scenarios *in vivo*. In a proof of concept approach, we evaluated the administration of Iloprost in a mouse osteotomy model system. Iloprost was inserted into a fibrin clot delivery system, from which the prostacyclin analog was successively released, which was shown by a downregulation of a pro-inflammatory cellular distribution around the fracture gap 3 days post osteotomy. The *in vivo* study confirmed the positive effect of Iloprost on bone regeneration. Animals treated with Iloprost showed a significantly improved bone healing outcome 21 days post-osteotomy compared to untreated control mice evaluated by μCT as well as histomorphometry after Movat's Pentachrome staining. The observed significantly enhanced bone formation in the Iloprost treated animals can be explained by an earlier starting of the regenerative process due to the down-regulated pro-inflammatory phase at the beginning of the healing cascade in comparison to the PBS treated control mice. Analysis of the cellular distribution of specific immune cells 3 days post-osteotomy in the fractured bones showed a clear tendency toward a reduced pro-inflammatory and increased anti-inflammatory cellular phenotype and cytokine secretion profile, as already observed in the preceding *in vitro* study. Furthermore, no effect on the relative amount of osteoblasts and osteoclasts in and around the fracture gap were detectable after Iloprost treatment 3 days post-osteotomy *in vivo*. *In vitro*, we also showed that the secreted cytokine milieu of activated and either Iloprost or untreated T cells has a significant impact on the osteogenic capacity of MSCs cultured in osteoinductive media. Our results showed a compensatory effect by the Iloprost treatment on the potential anti-regenerative cytokine milieu produced by activated T cells. Thus, the pro-regenerative effect of Iloprost is indirectly mediated on MSCs/osteoblasts by changes in the functionality of (pro-inflammatory) effector T cells participating in the bone regeneration cascade. Next to the effect of Iloprost on CD8+ T cells and macrophages, an influence on other immune cell could further impact bone healing *in vivo*. The early fracture healing phase is characterized by an infiltration of cells of the innate immunity like mast cells and neutrophils. For both, it was already shown, that the administration of prostacyclin analogs led to a reduced recruitment of these cells to the site of injury (34–36). Furthermore, an inhibitory function of Iloprost is reported for the secretion of effector cytokines of bone marrow dendritic cells as well as Th1 and Th2 CD4+ T cells *in vitro* (37). Thus, Iloprost is not only affecting the CD8+, but also the CD4+ T cell compartment in diminishing the (pro-inflammatory) effector cytokine secretion and thus function. One major key element of the potential of Iloprost to improve bone regeneration seems to be the time point of administration during the healing scenario. The group of Dogan reported an inhibitory effect of Iloprost on fracture repair in rats (38). There, Iloprost was administered by a daily injection for 5 days, starting at the time point of surgery. Due to the immune modulatory effect of Iloprost, a too early application of the drug could lead to an inhibition of the indispensable early pro-inflammatory phase initiating the healing cascade. This hypothesis is further supported by the reported effects of Iloprost on mast cells and neutrophils. Both cell types are indispensable for the early fracture healing phase to initiate the healing cascade. If Iloprost is administered directly at the time point of surgery/injury, it will inhibit the crucial recruitment of both cells types and will therefore disturbed the formation of the necessary cellular and cytokine milieu for a correct progression of the regeneration cascade (35, 36). Therefore, we used a fibrin clot for a (1) delayed and (2) successive release of Iloprost into the fracture gap thereby allowing the first pro-inflammatory phase to proceed. The positive impact of Iloprost in bone healing was already demonstrated in a small case study in the clinic for the treatment of subchondral stress fractures of the knee. Patients receiving Iloprost showed an improved healing of the stress fractures in comparison to the control group receiving the opioid analgesic Tramadol (39). Even though Iloprost is beneficial for bone healing applied systemically, this also bears considerable risks which would be circumvented by a local application as we proved in our proof of concept *in vivo* study. Thus, also in the treatment of patients, the possible pro-regenerative effect of Iloprost in bone healing was confirmed further supporting our *in vivo* and *in vitro* results.

## Conclusion

In the here presented study, the anti-inflammatory impact of Iloprost was confirmed. Cellular components of the immune system play a key role in bone fracture healing. An overwhelming pro-inflammatory phase in the early fracture healing cascade is correlated with an impaired healing outcome (15). We demonstrated that Iloprost has the potential to compensate the partial unfavorable pro-inflammatory effect of effector T cells and is able to stimulate the formation of an anti-inflammatory and pro-regenerative cellular milieu improving fracture healing. In a consecutive step, this strategy has to be confirmed in clinical phase I/II trials.

## Author Contributions

SW, KS-B, GD, and H-DV: conceptual idea and design of the study. SW, CS, CB, CJS, and JB: data collection, analysis, and interpretation. SW, CS, CB, KS-B, GD, and H-DV: drafting of the manuscript. All authors revised the manuscript.

### Conflict of Interest Statement

The authors declare that the research was conducted in the absence of any commercial or financial relationships that could be construed as a potential conflict of interest.
