# Supplementary material for: Immune Modulation to Enhance Bone Healing—A New Concept to Induce Bone Using Prostacyclin to Locally Modulate Immunity
Source: Front Immunol. 2019 Apr 5;10:713. doi: 10.3389/fimmu.2019.00713 (PMC6459956; doi:10.3389/fimmu.2019.00713)
Supplement: Supplementary file 1 [file Image_1.pdf]

Figure S1: Metabolic activity of activated and Iloprost treated CD8+ T cells, respectively

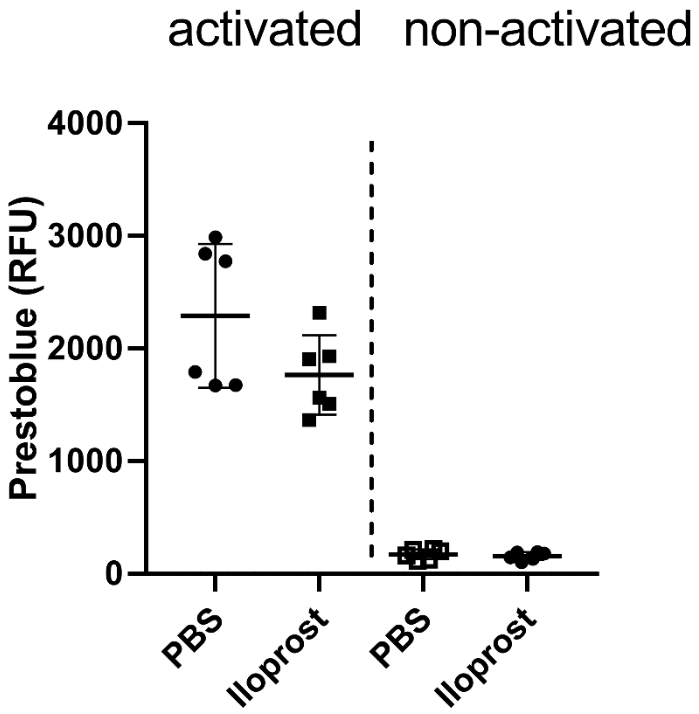

Figure S1: Metabolic activity of activated and Iloprost treated CD8+ T cells. Shown is the metabolic activity measured by Prestoblue. n = 6
